# Maximizing species conservation in continental Ecuador: a case of systematic conservation planning for biodiverse regions

**DOI:** 10.1002/ece3.1102

**Published:** 2014-05-17

**Authors:** Janeth Lessmann, Jesús Muñoz, Elisa Bonaccorso

**Affiliations:** 1 Centro de Investigación en Biodiversidad y Cambio Climático, Universidad Tecnológica Indoamérica, Machala y SabanillaCotocollao, Quito, Ecuador; 2 Universidad Internacional Menéndez PelayoIsaac Peral 23, Madrid, 28040, Spain; 3 Real Jardín Botánico (RJB-CSIC)Plaza de Murillo 2, Madrid, 28014, Spain; 4 Biodiversity Institute, University of KansasLawrence, 28014, Kansas

**Keywords:** Conservation feasibility, conservation priorities, Marxan, Maxent, protected area design, species distribution models, species diversity

## Abstract

Ecuador has the largest number of species by area worldwide, but also a low representation of species within its protected areas. Here, we applied systematic conservation planning to identify potential areas for conservation in continental Ecuador, with the aim of increasing the representation of terrestrial species diversity in the protected area network. We selected 809 terrestrial species (amphibians, birds, mammals, and plants), for which distributions were estimated via species distribution models (SDMs), using Maxent. For each species we established conservation goals based on conservation priorities, and estimated new potential protected areas using Marxan conservation planning software. For each selected area, we determined their conservation priority and feasibility of establishment, two important aspects in the decision-making processes. We found that according to our conservation goals, the current protected area network contains large conservation gaps. Potential areas for conservation almost double the surface area of currently protected areas. Most of the newly proposed areas are located in the Coast, a region with large conservation gaps and irreversible changes in land use. The most feasible areas for conservation were found in the Amazon and Andes regions, which encompass more undisturbed habitats, and already harbor most of the current reserves. Our study allows defining a viable strategy for preserving Ecuador's biodiversity, by combining SDMs, GIS-based decision-support software, and priority and feasibility assessments of the selected areas. This approach is useful for complementing protected area networks in countries with great biodiversity, insufficient biological information, and limited resources for conservation.

## Introduction

Protected areas are among the most effective strategies to reduce global biodiversity loss, and are central to almost all conservation policies (Glowka et al. 1994; Dudley and Parish 2006; Possingham et al. 2006). Protected area networks should be representative, meaning that all relevant biodiversity targets (e.g., species, ecosystems, etc.) are adequately accounted for, and protected within, the network (Pressey et al. 1993; Pressey 1994; Margules and Pressey 2000; Possingham et al. 2006). Despite the importance of this representativeness, only half of the terrestrial ecoregions have more than 10% of their extent protected (United Nations 2010). Moreover, several studies reveal large gaps of species representation in the current global network of protected areas (Rodrigues et al. 2004a,b2004b). Part of the reason is that many systematic planning decisions are made *impromptu* or opportunistically (Pressey 1994). Consequently, many protected areas are located in sites of relatively low economic and biodiversity values and, therefore, do not sustain an adequate representation of biodiversity (Margules and Pressey 2000).

In response to these problems and the limited resources available for conservation, research efforts have focused on the development of *systematic conservation planning*. This relatively new approach provides clear, comprehensive guides, to define efficient, representative, and complementary protected area networks (Pressey et al. 1993; Margules and Pressey 2000; Possingham et al. 2006). Within systematic conservation planning, decision-support software is applied to assist the selection and design of new protected areas (Margules and Pressey 2000). These algorithms identify sets of conservation areas under the *minimum-set problem* framework, which seeks to minimize resources expenditure, subject to the constraint that all biodiversity interests are represented adequately (Margules and Pressey 2000).

Ideally, to identify sets of suitable sites for protection, selection algorithms rely on complete, high-quality information on the spatial distribution of biodiversity indicators within a region (Carvalho et al. 2010). In practice, it is difficult to compile this information comprehensively, assuring both spatial and taxonomic representation (Carvalho et al. 2010). Specifically, when species are used as indicators of biodiversity, information on their distribution is often incomplete and spatially biased (Gaston and Rodrigues 2003). This situation is frequent in regions where systematic conservation planning and prioritization are more relevant, such as tropical countries with high biodiversity, high deforestation rates, and incipient protected area systems (Gaston and Rodrigues 2003). An alternative to using potentially biased species geographic information is to generate estimator surrogates, such as species distribution models (SDMs), which relate species occurrences to a set of geographic or environmental predictors (Elith and Leathwick 2009a). The integration of SDMs and site-selection algorithms constitutes a powerful tool for reserve design in regions with high biodiversity, where protection of areas of high diversity at the lowest cost, is urgent (Pawar et al. 2007; Elith and Leathwick 2009a).

This is the case of Ecuador, one of the 17 megadiverse countries, and the most biodiverse when considering species number by unit area (Sierra et al. 2002). Despite being a small country (284,000 km^2^), Ecuador has 44 state-protected areas that cover approximately 19% of its territory (continent and islands) (ECOLAP & Ministerio del Ambiente del Ecuador 2007). This situation positions it as one of the Latin American countries with the most area under some type of protected status (Elbers 2011). Unfortunately, this protected area network shows large conservation gaps for species and ecosystems (Sierra et al. 2002; Cuesta-Camacho et al. 2006). Also, in addition to a history of habitat changes that date back to pre-Columbian times, Ecuador is experiencing accelerated landscape fragmentation and degradation (Sáenz and Onofa 2005). The result is a dramatic reduction in natural vegetation cover (over 55%, SENPLADES 2009). More than 2200 species are listed as endangered because of habitat destruction, and illegal or indiscriminate harvesting (IUCN 2011). This great number of endangered species does not necessarily result from Ecuadorian species being particularly susceptible, but rather from sustained efforts directed to inventory species diversity in the country (Feeley and Silman 2011) and assessing their conservation status.

Given the high value, conservation gaps, and vulnerability of biodiversity in Ecuador, it is important to propose implementation of new nature reserves that complement the current protected area network. This need has been acknowledged by the Ecuadorian Government, which decided to increase the extent of this network by 5% (SENPLADES 2009). In this context, the use of reserve selection algorithms would be a valuable tool to guide the efficient allocation of the scarce resources available for protecting biodiversity.

Previous studies have identified global priority areas for conservation using species or ecosystems as indicators (Olson and Dinerstein 1998; Myers et al. 2000; Rodrigues et al. 2004b). However, global approaches cannot provide sufficiently detailed information to guide the establishment of new protected areas in specific countries (Sierra et al. 2002). To date, only two studies have focused on the identification of priority areas in continental Ecuador, and both used ecosystems as main biodiversity indicators (Sierra et al. 2002; Cuesta-Camacho et al. 2006). Thus, complementary studies to identify areas that ensure species representation are crucial for conservation planning, as species are considered fundamental in the evolution of biodiversity, and constitute essential indicators for monitoring the status of global biodiversity (Convention on Biological Diversity 2010).

In this study, we combined the use of SDMs and a reserve selection algorithm to identify new and complementary potential areas for species conservation in continental Ecuador. To accomplish this aim, we (1) selected the target species and compiled input data; (2) estimated the species distributions using SDMs; (3) set quantitative conservation targets for each species; (4) measured the conservation targets achieved by the current protected area network; (5) selected additional conservation areas to achieve the defined species conservation goals; and (6) evaluated the conservation priority and feasibility of each proposed area. As such, we seek to provide practical methodological guidance in reserve selection exercises, which may contribute to improve protected area networks in Ecuador and other highly diverse countries.

## Methods

### Study area

**Figure 1:**
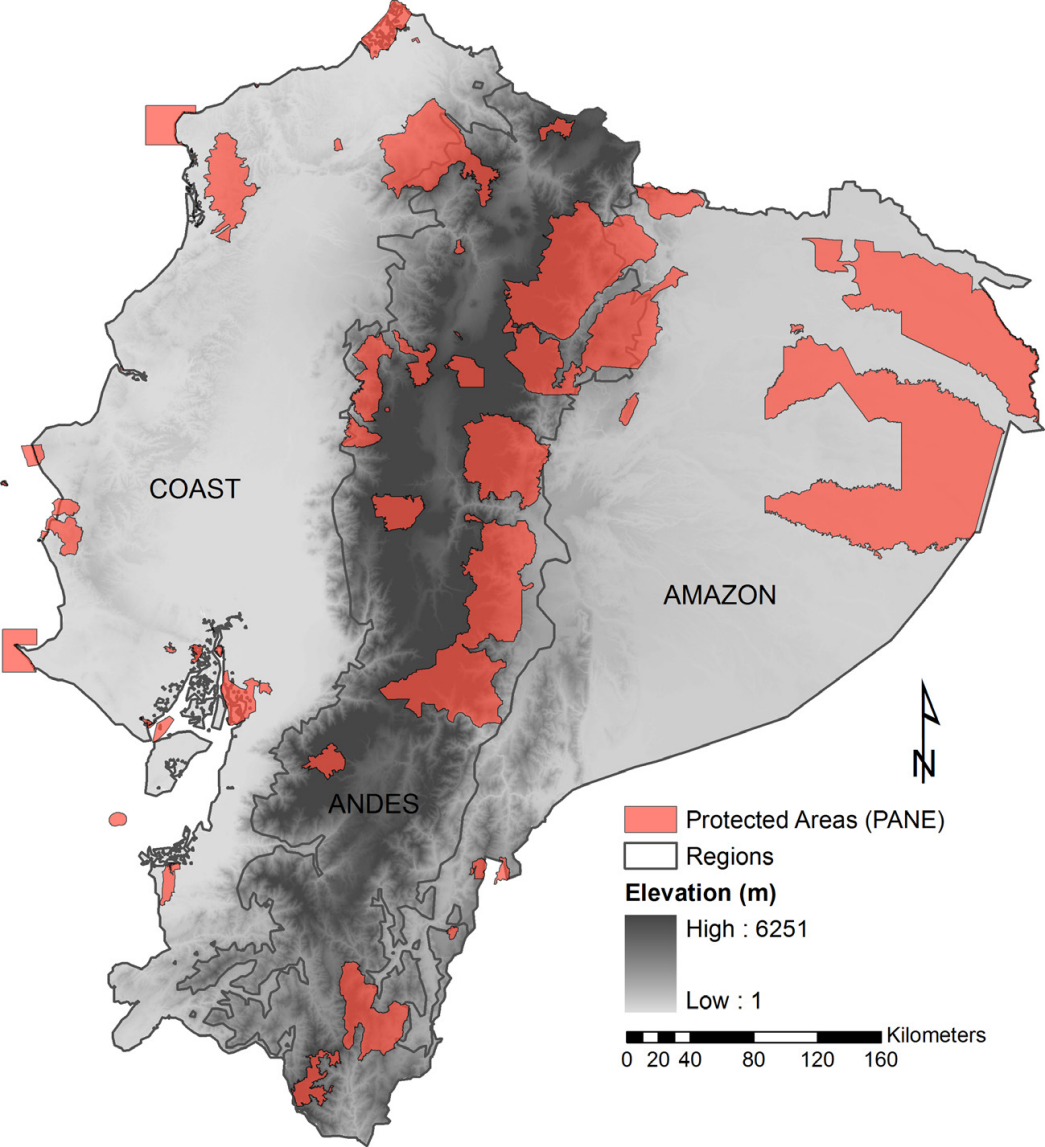
Current protected areas and regions of continental Ecuador.

Our planning exercise was conducted in continental Ecuador, which corresponds to 96.62% (248,313 km^2^) of the country's total landmass. Continental Ecuador is usually classified into three regions: Coast, Andes, and Amazon (Fig. 1). The Coast comprises territories below 1300 m in the western foothills of the Andes; its main ecosystems are coastal rainforests, dry forests, and mangroves (Sierra et al. 1999). The Andean region covers areas from 1300 m to the top of the mountains (∼6313 m) and includes montane wet forest, moorland, and wet and dry inter-Andean vegetation (Sierra et al. 1999). The Amazon region lies below 1300 m in the eastern foothills of the Andes and includes Amazonian rain forest and flooded (*varzea*) forests, among others (Sierra et al. 1999).

There are 41 state-protected areas in continental Ecuador included in the “*Patrimonio de Áreas Naturales del Estado*” (PANE), which covers approximately 16.5% of the territory (Cuesta-Camacho et al. 2006). In addition to this national system, there are other areas with different levels of protection, including the publicly or privately owned protected forests (“*Bosques Protectores*”), established to protect watersheds and where sustainable development is allowed. Protected areas within PANE, protected forests, and other publicly or privately owned areas constitute the National System of Protected Areas, SNAP (ECOLAP & Ministerio del Ambiente del Ecuador 2007). Herein, we looked for potential conservation areas that complement the biodiversity preserved only by PANE, as these reserves are more likely to persist over time.

### Conservation targets

To select the conservation targets, we focus on the best-studied species groups in Ecuador, which correspond to birds, amphibians, mammals, and vascular plants. For these species, there are more complete inventories across the country, as well as available references about their taxonomy and distribution. Specifically, we selected 809 terrestrial species: 182 amphibians, 69 birds, 52 mammals, and 506 plants that had enough occurrence records in Ecuador to generate reliable SDMs. These records were compiled from specimen databases, including species in different levels of threat (endangered–least concern), as well as endemic and widespread species (Appendix S1 in Supporting Information).

### Species distribution models

Given the lack of complete information about the distribution of target species, we used SDMs as an intermediate step toward estimating their geographic distributions. Species distribution modeling is a technique used to estimate potential areas of distribution, on the basis of observed presences and (sometimes) absences (Elith and Leathwick 2009b). To construct SDMs, we used Maxent, a machine-learning technique based on the principle of maximum entropy (Phillips et al. 2006; Elith et al. 2011; Renner and Warton 2013). As predictor variables, we used presence data and the 19 bioclimatic variables from Worldclim 1.4, a set of global climate layers derived from precipitation and temperature at ∼1 km^2^ spatial resolution (http://www.worldclim.org; Hijmans et al. 2005). Models were developed with Maxent 3.3.3e, setting the convergence threshold to 10^−5^, maximum iterations to 500, and the regularization parameter to “auto” (see Appendix S2 “Extended Methods” in Supporting Information).

### Species conservation goals

We defined species conservation goals as the proportion of the species distributions (obtained from the SDMs) to be included by the site-selection algorithm in the protected area network. Specific proportions of species distributions to protect were selected based on extinction risk, taxonomic uniqueness, geographic extension, and dispersion home range of the species (see Appendix S2 “Extended Methods” in Supporting Information). We established conservation goals between 15% and 30% of the species distribution, as estimated from the corrected SDMs areas. The specific conservation goals for each species were obtained in a linear scale from 15% (assigned to species with the lowest conservation priority), up to 30% (assigned to species with the highest priority). This assignation was based on preliminary analyses, indicating that species conservation goals above 30% require conservation areas that exceed a viable size for the economic and environmental reality of Ecuador.

### Representativeness of currently protected areas

We studied how the proposed species conservation goals were achieved by the current system of protected areas, by calculating the proportion of the extent of the species distributions contained within current protected areas. Two types of conservation gaps were identified: (1) when species distributions lie entirely outside of the protected area system and (2) when the species exists within the system, but with insufficient area to achieve the species conservation goals (Dudley and Parish 2006). Gap analyses were conducted by species taxonomic group, geographic region (Coast, Andes, Amazon), and extinction risk category.

### Identifying potential areas for conservation

We used the Marxan algorithm (Ball et al. 2009) to select areas that together represent the diversity of species in the most efficient manner. We called them potential areas for conservation (PACs). In each of multiple iterations, Marxan identifies a set of planning units (PUs) that meets predefined species conservation goals while minimizing the “total cost” of the solution (Ardron et al. 2008; Game and Grantham 2008). The “total cost” is defined as:


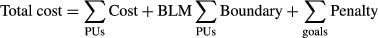


where summation of PUs cost represents some measure of the cost of including a PU; summation of PUs boundary is a cost related to the length of the solution perimeter (agglomerate vs. disperse solution, tuned with the boundary length modifier, BLM); and the summation of species conservation goal penalty is the costs imposed for failing to meet conservation goals.

**Figure 2:**
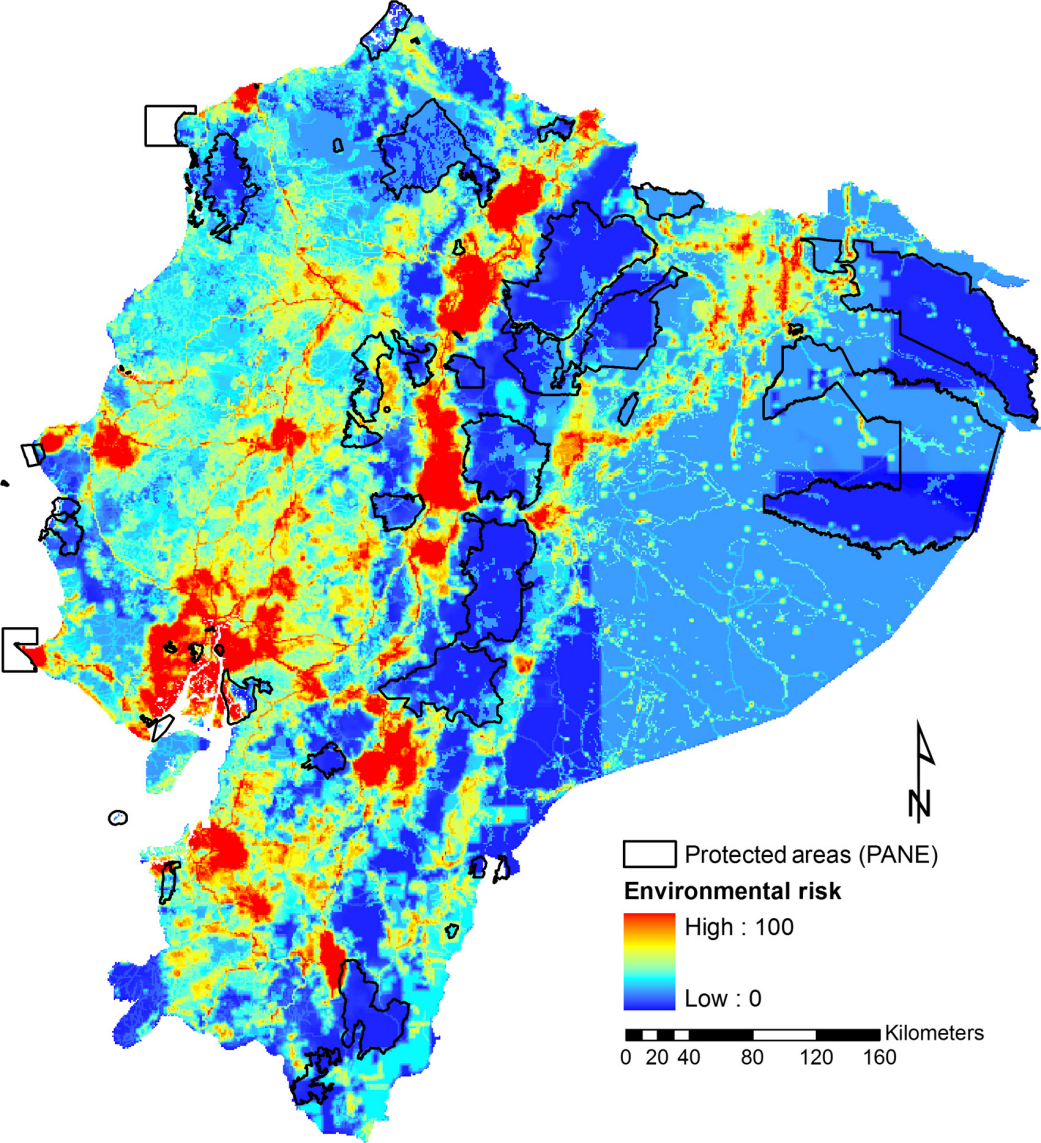
Environmental risk surface for continental Ecuador. This surface was generated from geographic information on roads, human population density, airports, dams, agriculture and husbandry, and oil and mining industry.

Herein, cost will be a synonym of environmental impact, because areas with higher environmental degradation are less suitable for conservation and should carry a higher cost of inclusion in the reserve network. To determine the level of environmental impact for each PU, we developed an Environmental Risk Surface (ERS, Fig. 2, Appendix S2 “Extended Methods” in Supporting Information) using as input different maps of human threats and the software *Protected Area Tools* for ArcMap 9.2 (http://gg.usm.edu/pat/) (McPherson et al. 2008). We also used a BLM of 0.01, which allows high connectivity between planning units in the solution, and assigned penalty factor (PF) values between 0.1 and 1 for each species, to increase the likelihood of achieving the species conservation goals.

Planning units for continental Ecuador were 50,507 hexagons of 5 km^2^. We forced all the PUs belonging to existing protected areas to be included in all solutions. This procedure allows considering the proportion of species distributions already protected; hence, the final portfolio is made of new areas that complement the current protected areas. Also, we excluded all PUs with the highest convergence of risk elements (>65 impact value) and PUs completely covered by transformed vegetation, to avoid their selection in the solution.

We executed Marxan with the simulated annealing method, followed by iterative improvement, using 10^8^ iterations and 100 runs. We analyzed two sets of results: the best solution and the summed solution. The best solution is the run with the lowest total cost and therefore the most efficient of the 100 runs (Game and Grantham 2008). The summed solution provides the frequency in which each PU is selected across the 100 runs and therefore indicates how important a PU is for creating an efficient reserve system (Ardron et al. 2008).

### Conservation priority and feasibility

As insufficient resources are available to implement the entire network of reserves selected by Marxan, we evaluated each PAC using priority and feasibility of protection to select the best set of areas for biodiversity conservation (see Appendix S2 “Extended Methods” in Supporting Information). Priority equals the urgency to protect an important site for biodiversity that may experience high environmental vulnerability. We evaluated this criterion using two variables: (1) importance of the area to achieve an efficient reserve system, calculated as the selection frequency of each PU (summed solution) in each selected area and (2) environmental impact of the area (from ERS). We summed the values of these two variables to determine the conservation priority of the each PAC, as maximum priority (areas with high importance and high environmental impact), high priority (low importance but high environmental impact), medium priority (high importance but low environmental impact), and low priority (low importance and low environmental impact).

Conservation feasibility equals the opportunity of successfully implementing an area as a suitable reserve for the persistence of biodiversity. The feasibility assessment was also conducted using two variables: (1) previous conservation efforts carried in a selected area (i.e., proportion of the potential conservation area within protected forests and buffer zones of 10 km around reserves) and (2) proportion of remaining natural vegetation in the potential conservation area, as areas with low proportion of remaining natural vegetation require ecological restoration, demanding higher economic investments. The values obtained by each area were classified in four groups: maximum feasibility (high proportion of land within protected forests and buffer zones, and high proportion of natural vegetation), high feasibility (low proportion of land within protected forest and buffer zones, but high proportion of natural vegetation), medium feasibility (high proportion of land within protected forests and buffer zones, and low proportion of natural vegetation conserved), and low feasibility (low proportion of land within protected forests and buffer zones, and little natural vegetation remaining).

## Results

**Figure 3:**
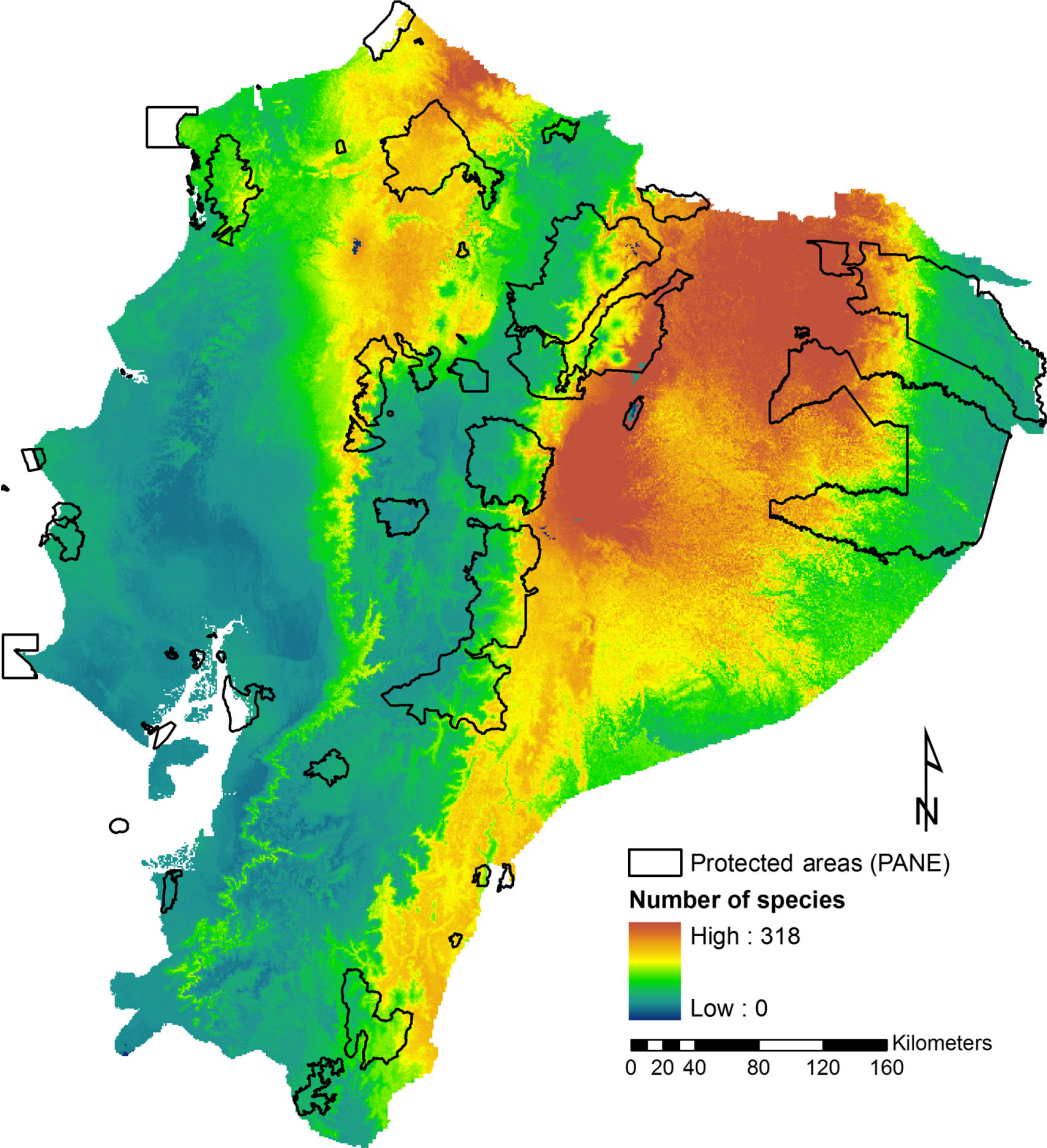
Potential biodiversity richness for continental Ecuador. The diversity map includes the 809 species used as conservation target in this study.

Based on the ensemble of 809 SDMs, the highest diversity for all species is located in the northern Amazon and the northern coast of Ecuador or Ecuadorian Chocó (Fig. 3). This pattern agrees with previous studies at the continental scale that included Ecuador (Myers et al. 2000; Bass et al. 2010).

### Achievement of species conservation goals by the current protected area network

The current protected area network achieved the defined conservation goals for 309 species (38.2%, Table 1). Of the remaining 500 species (61.8%), 4 (0.5%) are completely out of the PANE and 496 (61.3%) are insufficiently protected. Only mammals, bromeliads, and moorland plants have more than 50% of their species with conservation goals achieved. Among the endangered species (CR, EN, VU), only 26.9% achieved their conservation goals. Species of the Coast are the least protected; only 17.3% achieved their conservation goals, compared with 41.8% and 40.3% of the species in the Andes and Amazon, respectively.

**Table 1:** Goal achievement in current protected areas of continental Ecuador, according to species group, extinction risk, and geographic region

Category		No. of species with goals achieved	No. of species with missed goals
Species group	Amphibians	53 (29.1%)	129 (70.9%)
	Birds	17 (24.6%)	52 (75.4%)
	Mammals	27 (51.9%)	25 (48.1%)
	Araceae	23 (43.4%)	30 (56.6%)
	Bignoniaceae	1 (9.09%)	10 (90.9%)
	Bromeliaceae	43 (55.8%)	34 (44.6%)
	Gesneriaceae	37 (46.3%)	43 (53.8%)
	Lauraceae	9 (34.6%)	17 (65.9%)
	Leguminous	18 (18%)	82 (82%)
	Moorland plants	17 (100%)	0 (0%)
	Rubiaceae	64 (45.1%)	78 (54.9%)
	Total	309 (38.2%)	500 (61.8%)
Extinction risk	CR	6 (22.2%)	21 (77.8%)
	EN	12 (23.5%)	39 (76.5%)
	VU	22 (31%)	49 (69%)
	NT	17 (37.8%)	28 (62.2%)
	NE	205 (41.7%)	293 (58.8%)
	DD	7 (28%)	18 (72%)
	LC	40 (43.5%)	52 (56.5%)
Region	Coast	57 (17.3%)	273 (82.7%)
	Andes	137 (41.8%)	191 (58.2%)
	Amazon	190 (40.3%)	281 (59.7%)

Similar to the study by Cuesta-Camacho et al. (2006), we found that 16.3% of the terrestrial areas in continental Ecuador are protected by PANE (Table 2). The Coast has only 5.1% of its territory protected by PANE, compared with 20.6% for the Andes and 23.2% for the Amazon (Table 2). A large proportion of areas of great diversity (Chocó region and part of northern Amazon) lack formal protection. Large conservation gaps found in the current protected area network indicate that it is necessary to propose a significant increase in the reserve system to meet the unachieved conservation goals. On average, current protected areas recorded environmental impacts of 22.72 (± 29.69). These values are low when compared to the impact records of 50.93 (± 41) outside protected areas.

**Table 2:** Terrestrial extension of continental Ecuador included in PANE and the potential areas for conservation by geographic region

	Extension protected by PANE		Extension protected by the PACs		Extension protected by the PACs and PANE	
			
Region	km^2^	%	km^2^	%	km^2^	%
Coast	4151.83	5.1	19,128	23.6	23,280	28.7
Andes	18,288.50	20.6	15,562	17.5	33,850	38.1
Amazon	18,091.30	23.2	15,153	19.4	33,245	42.6
Continental Ecuador (total)	40,532	16.3	49,843	20.1	90,375	36.4

### Identifying potential areas for conservation

The best solution chosen by Marxan was used to define the areas with potential to be included in the current protected area network. In this solution, Marxan reached the conservation goals for 802 species; only seven showed low (7–12%) conservation goal deficits, because most of their distributions fall into highly transformed lands, previously excluded from the solution.

**Figure 4:**
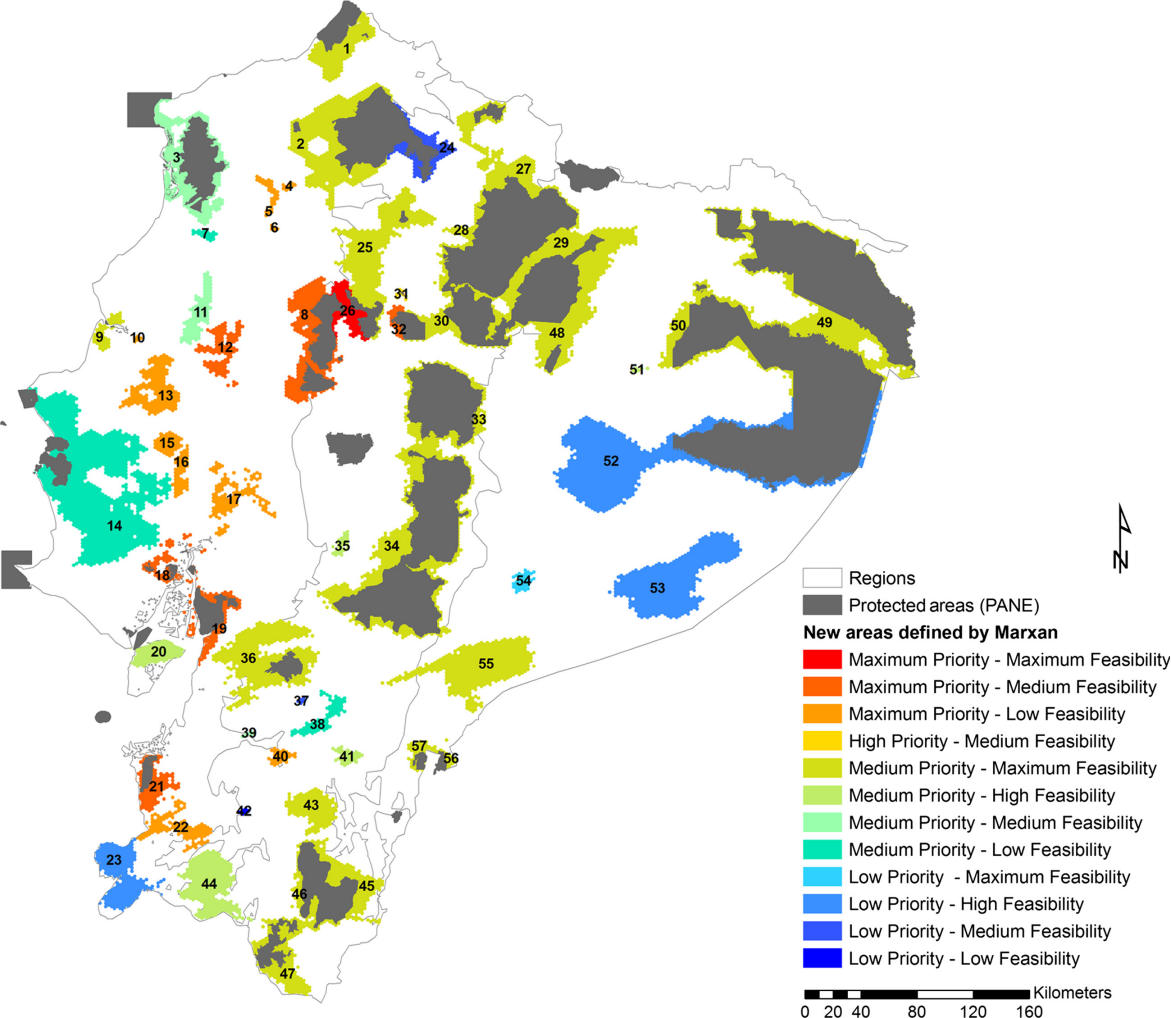
Potential protected areas for the conservation of terrestrial species in continental Ecuador. The potential conservation areas were chosen from the first best solution in Marxan, with indication of its classification according to the priority and feasibility assessments. (1) Extension of Manglares Cayapas Mataje ER, (2) corridor between Cotacachi Cayapas ER and El Pambilar WR, (3) corridor between Mache Chindul ER and Ecosistema de Manglar del Estuario del Río Mindo ER, (4–6) new areas in the northern Coast, (7) new area in the south of Mache Chindul ER, (8) extension of the western Los Ilinizas ER, (9) extension of Islas Corazón y Fragatas WR, (10–13) new areas in the central Coast, (14) corridor between Machalilla NP and Pacoche WR, (15–17) new areas in the central Coast, (18) corridor between Manglares el Salado WPR and Parque Lago NRA, (19) extension of Manglares Churute ER, (20) new area in Puná Island, (21) extension of Arenillas ER, (22) new area in the southern Coast, (23) new area in the south end of the Coast, (24) extension of the eastern Cotacachi Cayapas ER, (25) corridor between Pululahua GR and Los Ilinizas ER, (26) corridor between Los Ilinizas ER, (27) corridor between El Ángel ER and Cayambe Coca NP, (28) extension of the western Cayambe Coca NP, (29) corridor between Cayambe Coca NP, Sumaco Napo Galeras NP, and Antisana ER, (30) corridor between Cotopaxi NP and Antisana ER, (31) extension of Pasochoa WR, (32) extension of the western Cotopaxi NP, (33) extension of Llanganates NP and corridor between Sangay NP, (34) extension of Sangay NP, (35) new area in the central Andes, (36) extension of Cajas NP, (37–43) new areas in the southern Andes, (44) new area in the south end of the Andes, (45) extension of Podocarpus NP, (46) corridor between Podocarpus NP and Yacurí NP, (47) extension of Yacurí NP, (48) extension and corridor within Sumaco Napo Galeras NP, (49) corridor between Cuyabeno WPR, Limoncocha BR and Yasuní NP, (50) extension of Yasuní NP, (51) new area in the northern Amazon, (52) extension of the southern Yasuní NP, (53) New area in the east end of the Amazon, (54) new area in the north of Cutucú mountain range, (55) new area in the Cordillera Cutucú mountain range, (56) extension of El Cóndor BR, (57) corridor between El Cóndor BR and El Quimi BR. ER, ecological reserve; WR, wildlife refuge; NP, national park; WPR, wildlife production reserve; NRA, national recreation area; GR, geobotanical reserve; BR, biological reserve.

Planning units selected in the best solution were grouped within 57 PACs (Fig. 4), of which the highest proportion is located in the Coast (Table 2). These 57 areas constitute extensions of current protected areas, biological corridors among currently existing areas, or entirely new areas. Potential areas for conservation and current protected areas cover 90,375 km^2^, which represents 36.4% of the continental territory of Ecuador. Therefore, implementing this proposed network would imply doubling the extent of the current system of protected areas (from 16.3% to 36.4% of continental Ecuador).

### Conservation priority and feasibility of the potential conservation areas

From the total of 57 PACs, we identified 17 of maximum conservation priority; 14 of them located in the Coast and three in the Andes. Of the remaining areas, one is classified as high priority, 32 as medium priority, and seven as low priority. In terms of feasibility, 23 areas have maximum feasibility of establishment, eight have high feasibility, 12 have medium feasibility, and 14 have low feasibility. Thirteen of the most feasible areas are located in the Andes, seven in the Amazon, and three in the Coast. Of the least feasible areas, 11 are in the Coast and three in the Andes. Most areas with high priority have low feasibility, while areas with high feasibility have medium priority. A summary of priority/feasibility of areas and their extents is provided in Fig. 4 and Table 3.

**Table 3:** Extent of protected areas proposed for continental Ecuador according to each category of priority–feasibility of conservation

	Feasibility				
	
Priority	Maximum	High	Medium	Low	Subtotal
Maximum	502 km^2^	0 km^2^	3248 km^2^	3195 km^2^	6945 km^2^
	1%	0%	6.5%	6.4%	14%
High	0 km^2^	0 km^2^	34 km^2^	0 km^2^	34 km^2^
	0%	0%	0.1%	0%	0.1%
Medium	23987 km^2^	2165 km^2^	6143 km^2^	1838 km^2^	34133 km^2^
	48.1%	4.3%	12.3%	3.7%	68%
Low	180 km^2^	7980 km^2^	541 km^2^	30 km^2^	8731 km^2^
	0.4%	16%	1.1%	0.1%	18%
Subtotal	24669 km^2^	10145 km^2^	9965 km^2^	5063 km^2^	49843 km^2^
	49.5%	20.4%	20%	10.2%	100%

## Discussion

Ecuador, as many other tropical countries, faces significant challenges to define new protected areas because of its great biodiversity, scarce biological information, and limited resources for conservation. Despite these constraints, our combined use of SDMs, site-selection algorithms, and priority and feasibility conservation analysis provides a powerful tool for selecting new areas that will contribute to preserve Ecuador's astonishing biodiversity. To get robust results, we used a large set of conservation targets (809 species), including plants and three groups of vertebrates, representing different categories of threat and levels of endemism. We expect to find more representative areas than studies using only one taxonomic group or other indicators, such as endangered, charismatic, or umbrella species. Thus, we assessed the degree to which terrestrial species diversity is currently being protected, identified potential conservation areas that better achieve predefined conservation goals, and assessed the priority and feasibility of conservation of these new areas.

### How well represented is Ecuador's species diversity within the current reserve system?

The current protected area network of continental Ecuador showed large conservation gaps for the species analyzed. Almost all species have part of their distribution within reserves, but most of them have a low proportion of that distribution protected. According to the species conservation goals, the great majority of species (61.8%) are insufficiently protected. Only species of moorland plants fully achieved their conservation goals, mainly because they are distributed across the Andes, the richest region in reserves. Most of the endangered species (∼75%) were insufficiently represented by the current protected area network. Likely, their restricted geographic ranges decrease the chance that their distributions overlap with protected areas.

Considering the three regions of continental Ecuador, the Coast showed the largest conservation gaps. Historically, this region has supported much of the country's agriculture and has suffered from significant urban development (Sierra et al. 1999; Sáenz and Onofa 2005). This situation has been an obstacle for the establishment of protected areas, especially in the central Coast, which is characterized by large spans of transformed land. In contrast, the Andean region showed the smallest conservation gaps, which resulted from the high concentration of large protected areas. However, 13 of the 16 Andean reserves are located in the northern Andes, which may result in conservation gaps for species and ecosystems with southern distributions. In the central Amazon (southern Napo and western Pastaza provinces), we observed both high diversity and lack of protection. As it happens in the Andes region, protected areas of the Amazon are located mostly in the north, leaving significant gaps in the south.

Despite the conservation gaps found for target species, the territories included in the current protected area network showed lower values of environmental impact compared with the rest of Ecuador. Thus, it may be concluded that existing protected areas are indeed playing an important role in preventing environmental degradation across Ecuador.

### Potential areas for conservation in continental Ecuador

Large conservation gaps in the Coast resulted in a major concentration of proposed areas along this region. Our study identified eight potential reserves for the northern Coast, encompassing the southern portion of the Chocó (Fig. 4: 1–8). This bioregion contains a high richness of target species and has been classified as a biodiversity *hotspot* because of its great diversity and high vulnerability (Myers et al. 2000).

The main conservation challenge is the central Coast, which has experienced extensive transformation of the original ecosystems by intensive agriculture (Sáenz and Onofa 2005). However, small patches of natural vegetation still remain in this heavily altered zone. We decided to include PUs with partially transformed vegetation that allows (1) connecting fragments of natural vegetation; (2) creating buffers to maintain natural vegetation; and (3) implementing easier habitat restoration, as transformed vegetation is closer to the source of native vegetation. Thus, habitat-degraded lands within potential conservation areas in the central Coast (such as 9–19 in Fig. 4) are good options for restoration programs.

The southeastern Coast (Tumbesian region), where we identified a high richness of bird species, is considered an important bird area (BirdLife International 2012b). Here, three PACs were selected (Fig. 4: 21–23), all adjacent to three protected areas in Peru (Cerros de Amopote National Park, Tumbes Wildlife Refuge, and El Angolo Hunting Reserve). Formal recognition of these areas in Ecuador may promote binational efforts for conservation of the unique Tumbesian avifauna. Similarly, PACs in the coastline (Fig. 4: 14) compose a geographic unit that plays a crucial role in climate regulation in the region and are considered of special interest for species protection, especially birds (BirdLife International 2011).

The Andes is the region where more species reached their conservation goals and, thus, the region with the lowest proposed increase in protected areas. However, our results suggest that several potential conservation areas are needed to complete the defined goals and create corridors increasing conservation efficiency of the current network. Specifically, the western Andes are under higher threat and harbor less protected areas than the eastern Andes. Consequently, the corridors between the Cotacachi Cayapas Ecological Reserve, Pululahua Geobotanical Reserve, and Los Illinizas Ecological Reserve (Fig. 4: 24–26) are especially relevant.

In the eastern Andes, the proposed areas (Fig. 4: 27–30, 32) comprise several corridors that connect five, mostly northern protected areas: El Angel Ecological Reserve, Cayambe Coca National Park, Sumaco Napo Galeras National Park, Antisana National Park, and Cotopaxi Ecological Reserve. There is also a central southern corridor between the Sangay National Park and Llanganates National Park (Fig. 4: 33), which might be important for the conservation of large-sized mammals and birds (Fonseca et al. 2003; BirdLife International 2012a). Finally, our study identified 12 areas in the southern Andes (Fig. 4: 36–47), which would level the current bias toward the northern Andes.

In the Amazon region, Marxan proposed corridors and extensions for the large protected areas Cuyabeno Wildlife Reserve, Yasuní National Park, and Sumaco Napo Galeras National Park (Fig. 4: 48, 49, 50 and 52). Also, as the southern and the eastern foothills of the Amazon are poorly represented in current protected areas, Marxan proposed new areas for protecting the southern Amazon (Fig. 4: 53–57).

Results using ecosystems as conservation targets may differ from the studies using species. For example, studies focused on ecosystems found that protected areas already existing in the north represent Amazon ecosystems adequately (Sierra et al. 2002; Cuesta-Camacho et al. 2006). However, from a species-based perspective, the high species richness in the northern Amazon is not adequately protected. Consequently, our results suggest that more conservation efforts are needed in the northern Amazon, to protect irreplaceable areas around Cuyabeno and Yasuní (Fig. 4: 49–52). Conversely, our results agree with two previous ecosystem-based studies (Sierra et al. 2002; Cuesta-Camacho et al. 2006) where the Coast is considered the region in most need of protection. Combining species- and ecosystem-based studies would improve the decision makers solution portfolio, avoiding biases and allowing better-informed decisions.

### Which areas should be protected first?

Several studies that propose new areas for conservation are limited to identify sites of importance, without analyzing their priority and feasibility of conservation. Such analysis is very useful as, frequently, it is difficult to implement all PACs as reserves. Indeed, in our work, the extension of PACs is more than twice the national protected area network. Therefore, it is more realistic to present an assessment of conservation priority and feasibility for the proposed areas, which may guide and support decision making.

The highest priority areas were located mainly in the Coast, where the transformation and degradation of natural ecosystems have been the fastest in Ecuador (Sierra et al. 1999). However, despite having high conservation priority, many areas in the Coast present low and medium feasibility. This situation results from the combination of high habitat degradation, small extension of protected forests, and few protected areas, reducing the chances of selecting areas with high feasibility in this region. In the Andes, the highest priority areas are located near urban centers and adjacent to zones transformed by agriculture and cattle farming. In addition, the opportunity costs of farming and grazing on the Coast are the highest in Ecuador (Naidoo and Iwamura 2007). Therefore, the implementation of the PACs identified for this region may require significantly high acquisition costs, because of foregone opportunities to use the land in economically valuable ways.

In contrast to the Coast and the Andes, the PACs in the Amazon were mostly considered of medium and low priority, because of the combination of low environmental impact and low importance. The low importance may derive from the fact that a large number of PUs in the Amazon are equally important to achieve the proposed conservation goals, and thus virtually interchangeable. However, despite the low priority and high feasibility of the Amazon areas, its biodiversity suffers from high vulnerability because over 65% of the territory is divided into blocks for oil exploitation (Finer et al. 2008). Regardless of the difficulty of implementing protected areas within oil blocks, our results highlight the importance of Amazon areas for biodiversity conservation.

Regarding the Government's goal of increasing the protected area to encompass an additional 5% of the Ecuadorian territory [20], it is clear that our results largely exceed the established increase. There are several aspects that play a role in deciding which of the PACs may be included in the SNAP. Depending on geographic location, the interested administration or private conservation organization should analyze the goals that need to be fulfilled in each case. Based on our analysis of feasibility and priority, protecting the corridor between the northern and central sections in which Los Ilinizas Ecological Reserve is divided (Fig. 4: 26), which represents 0.2% of the Ecuadorian territory, would be a good start, as it presented high priority and feasibility of conservation. The areas of highest priority and medium feasibility located in the Coast (Fig. 4: 8, 12, 18, 19, and 21) represent 1.3% of Ecuador and may well be a priority to achieve the goal. All these areas require a high economic investment for restoration, but they would preserve highly endangered biodiversity. Finally, protecting areas of maximum feasibility and medium priority (mainly because of their low impact), which represent 3.4% of Ecuador, would complete the Government's goal of 5%. The latter set includes six areas, which may be of interest according to the current conservation gaps (Fig. 4): Ecuadorian Chocó (1, 2), western Andean corridor (25), southern Andean slopes (43), the corridor between the Podocarpus and Yacurí national parks (46), and the Cutucú mountain range (55).

The conservation of areas with only a small proportion of their extension within protected forest or buffer zones may be also considered of high urgency, because although seemingly of low feasibility, they are important for complementing representativeness and are currently out of the protected area network. From this point of view, we highlight new areas with low protection in the Amazon (Fig. 4: 52–54), the Andes (Fig. 4: 35, 38, 40–42, 44), the southern (Fig. 4: 22 and 23), central (Fig. 4: 10, 13, 15–17) and northern (Fig. 4: 4–7) Coast, which summed compose 5.4% of Ecuador. Further options to reach the goals established by the Ecuadorian Government are the remnants of pristine land, characterized by low environmental impact and land transformation, in the Andes (Fig. 4: 30, 41 and 43) and the Amazon (Fig. 4: 49, 52, 53, 55–57), jointly representing 5.1% of Ecuador. However, using this last criterion, no protected area would be added in the Coast, the region with greatest conservation gaps.

We are aware that the establishment of new protected areas depends heavily on the management of socio-economic conflicts and in the knowledge that decision makers have about stakeholder's interests (Margules and Pressey 2000; Dudley and Parish 2006). It is equally important to consider the economic cost of the limitations imposed by conservation constraints, as the purpose of conservation planning is to efficiently achieve conservation objectives with limited resources (Naidoo et al. 2006). Therefore, for future projects, it is important to develop detailed cost maps that summarize acquisition, management, and opportunity costs (Naidoo et al. 2006). It is important to note that despite the absence of economic costs in our analysis, the priority areas that we identified are useful to guide the conservation planning in Ecuador given their efficiency in important aspects such as size, connectivity, low level of human intervention, species representativeness, and complementarity.

The current protected areas of Ecuador have several limitations regarding management, given budget deficits, and high pressure on land resource extraction (Galindo et al. 2005; Castaño-Uribe 2008). Therefore, in this context, it seems difficult to promote the establishment of new protected areas. However, private initiatives may assume the costs of establishing new conservation areas. In fact, private reserves have proved to be viable options for conservation in Ecuador and currently cover 4.6% of the country (Castaño-Uribe 2008; Elbers 2011).

### Final considerations

Our reserve design shows some limitations inherent to the tools used. For example, in some cases, SDMs may generate false species presences that would conduct to the design of nonrepresentative and inadequate reserve networks (Carvalho et al. 2010). However, other types of data available for highly diverse tropical areas, such as observed points or geographic ranges, usually produce more errors than SDMs (Rondinini et al. 2005; Carvalho et al. 2010). Also, the absence of persistence criteria for selecting potential conservation areas (i.e., population dynamics or climate change related shifts) was not considered here, as they were beyond the scope of this study. However, these and other limitations are likely to be minor compared with the urgency of taking informed conservation measures to slow the ongoing loss of biodiversity in tropical countries (Groves et al. 2002). Informed measures will improve the effectiveness of reserve networks and, ultimately, will contribute to better protection for biodiversity, in contrast with an *impromptu* selection of sites. Clearly, the task ahead involves improving data availability and the limitations inherent to conservation planning, in order to enable us to act strategically in the face of increasing human pressure (Rodrigues et al. 2004a).

## Supporting Information

**Appendix S1**. List of species used.

**Appendix S2**. Extended Methods.

## References

[b1] Ardron JA, Possingham HP, Klein CJ (2008). Marxan good practices handbook.

[b2] Ball IR, Possingham HP, Moilanen A, Wilson KA, Possingham HP, Watts ME (2009). Marxan and relatives: software for spatial conservation prioritisation. Spatial conservation prioritisation: quantitative methods and computational tools.

[b3] Bass MS, Finer M, Jenkins CN, Kreft H, Cisneros-Heredia DF, McCracken SF (2010). Global conservation significance of Ecuador's Yasuní National Park. PLoS ONE.

[b4] BirdLife International (2011). http://www.birdlife.org/datazone/sitefactsheet.php?id=14625.

[b5] BirdLife International (2012a). http://www.birdlife.org/datazone/sitefactsheet.php?id=14557.

[b6] BirdLife International (2012b). http://www.birdlife.org/datazone/ebafactsheet.php?id=47.

[b7] Carvalho SB, Brito JC, Pressey RL, Crespo E, Possingham HP (2010). Simulating the effects of using different types of species distribution data in reserve selection. Biol. Conserv.

[b8] Castaño-Uribe C (2008). Diagnóstico y situación actual de las áreas protegidas en América Latina y el Caribe, Informe Regional.

[b9] Convention on Biological Diversity (2010). http://www.cbd.int/sp/.

[b10] Cuesta-Camacho F, Peralvo M, Ganzenmüller A, Sáenz M, Novoa J, Riofrío G (2006). Identificación de vacíos y prioridades de conservación para la biodiversidad terrestre en el Ecuador continental.

[b11] Dudley N, Parish J (2006). Closing the gap. Creating ecologically representative protected area systems: A guide to conducting the gap assessment of protected area system for the Convention on Biological Diversity.

[b12] ECOLAP & Ministerio del Ambiente del Ecuador (2007). Guía del Patrimonio de Áreas Naturales Protegidas del Ecuador.

[b13] Elbers J (2011). Las áreas protegidas de América Latina: Situación actual y perspectivas para el futuro.

[b14] Elith J, Moilanen A, Wilson KA, Possingham HP, Leathwick J (2009a). The contribution of species distribution modelling to conservation prioritization. Spatial conservation prioritization. Quantitative methods and computational tools.

[b15] Elith J, Leathwick JR (2009b). Species distribution models: ecological explanation and prediction across space and time. Annu. Rev. Ecol. Evol. Syst.

[b16] Elith J, Phillips SJ, Hastie T, Dudík M, Chee YE, Yates CJ (2011). A statistical explanation of MaxEnt for ecologists. Divers. Distrib.

[b17] Feeley KJ, Silman MR (2011). The data void in modeling current and future distributions of tropical species. Glob. Change Biol.

[b18] Finer M, Jenkins CN, Pimm SL, Keane B, Ross C (2008). Oil and gas projects in the western Amazon: threats to wilderness, biodiversity, and indigenous peoples. PLoS ONE.

[b19] Fonseca RM, Carrera JP, Enríquez T, Lasso DO, Pinto M, Tello JS (2003). Identificación preliminar de un corredor ecológico para mamíferos entre los Paques Nacionales Llanganates y Sangay. Revista de la Pontificia Universidad Católica.

[b20] Galindo J, Calvopiña J, Baus C, Ayllón F, Vela S (2005). Análisis de necesidad de financiamiento del Sistema Nacional de Áreas Protegidas (SNAP) del Ecuador.

[b21] Game ET, Grantham H (2008). Marxan user manual: for marxan version 1.8.10.

[b22] Gaston KJ, Rodrigues ASL (2003). Reserve selection in regions with poor biological data. Conserv. Biol.

[b23] Glowka L, Burhenne-Guilmin F, Synge H (1994). A guide to the convention on biological diversity.

[b24] Groves CR, Jensen DB, Valutis LL, Redford KH, Shaffer ML, Scott JM (2002). Planning for biodiversity conservation: putting conservation science into practice. Bioscience.

[b25] Hijmans RJ, Cameron SE, Parra JL, Jones PG, Jarvis A (2005). Very high resolution interpolated climate surfaces for global land areas. Int. J. Climatol.

[b26] IUCN (2011). http://www.iucnredlist.org.

[b27] Margules CR, Pressey RL (2000). Systematic conservation planning. Nature.

[b28] McPherson M, Schill S, Raber G, John K, Zenny N, Thurlow K (2008). GIS-based modeling of Environmental Risk Surfaces (ERS) for conservation planning in Jamaica. J. Conserv. Plann.

[b29] Myers N, Mittermeier RA, Mittermeier CG, da Fonseca GAB, Kent J (2000). Biodiversity hotspots for conservation priorities. Nature.

[b30] Naidoo R, Iwamura T (2007). Global-scale mapping of economic benefits from agricultural lands: Implications for conservation priorities. Biol. Conserv.

[b31] Naidoo R, Balmford A, Ferraro PJ, Polasky S, Ricketts TH, Rouget M (2006). Integrating economic costs into conservation planning. Trends Ecol. Evol.

[b32] Olson DM, Dinerstein E (1998). The Global 200: priority ecoregions for global conservation. Conserv. Biol.

[b33] Pawar S, Koo MS, Ahmed MF, Chaudhuri S, Sarkar S (2007). Conservation assessment and prioritization of areas in Northeast India: priorities for amphibians and reptiles. Biol. Conserv.

[b34] Phillips SJ, Anderson RP, Schapire RE (2006). Maximum entropy modeling of species geographic distributions. Ecol. Model.

[b35] Possingham HP, Wilson KA, Andelman SJ, Groom MJ, Meffe GK, Carroll CR, Vynne CH (2006). Protected areas. Goals, limitations, and design. Principles of conservation biology.

[b36] Pressey RL (1994). Ad Hoc reservations: forward or backward steps in developing representative reserve systems?. Conserv. Biol.

[b37] Pressey RL, Humphries CJ, Margules CR, Vane-Wright RI, Williams PH (1993). Beyond opportunism: key principles for systematic reserve selection. Trends Ecol. Evol.

[b38] Renner IW, Warton DI (2013). Equivalence of MAXENT and poisson point process models for species distribution modeling in ecology. Biometrics.

[b39] Rodrigues ASL, Andelman SJ, Bakarr MI, Boitani L, Brooks TM, Cowling RM (2004a). Effectiveness of the global protected area network in the representing species diversity. Nature.

[b40] Rodrigues ASL, Akçakaya HR, Andelman SJ, Bakarr MI, Boitani L, Brooks TM (2004b). Global gap analysis: priority regions for expanding the global protected-area network. Biosci. Rep.

[b41] Rondinini C, Stuart S, Boitani L (2005). Habitat suitability models and the shortfall in conservation planning for African vertebrates. Conserv. Biol.

[b42] Sáenz M, Onofa Á (2005). Reporte de los ecosistemas terrestres ecuatoriano. Indicadores de biodiversidad para su uso nacional.

[b43] SENPLADES (2009). http://plan.senplades.gob.ec/presentacion.

[b44] Sierra R, Cerón C, Palacios W, Valencia R (1999). Propuesta preliminar de un sistema de clasificación de vegetación para el Ecuador continental.

[b45] Sierra R, Campos F, Chamberlin J (2002). Assessing biodiversity conservation priorities: ecosystem risk and representativeness in continental Ecuador. Landscape Urban Plann.

[b46] United Nations (2010). The millennium development goals report 2010.

