# Supplementary material for: Maximizing species conservation in continental Ecuador: a case of systematic conservation planning for biodiverse regions
Source: Ecol Evol. 2014 May 17;4(12):2410–22. doi: 10.1002/ece3.1102 (PMC4203289; doi:10.1002/ece3.1102)
Supplement: Supplementary file 2 — Appendix S2. Extended Methods. [file ece30004-2410-sd2.docx]

**Supporting Information**

Appendix S2 Detailed Methods

1. **Species data**

The records of species occurrence were obtained from specimen data bases of the following museums: Missouri Botanical Garden, American Museum of Natural History, Academy of Natural Sciences, British Museum of Natural History, California Academy of Sciences, Cornell University, Diego F. Cisneros-Heredia Collection (Universidad San Francisco de Quito), Museo Ecuatoriano de Ciencias Naturales, Delaware Museum of Natural History, Fundación Herpetológica G. Orcés, Florida Museum of Natural History, The Field Museum, Instituto de Biología - Universidad Nacional Autónoma de Mexico, Museo de Historia Natural Universidad Nacional de Colombia, Museum of Natural History University of Kansas, Natural History Museum of Los Angeles County, Museum of Natural Science of Louisiana State University, Museum of Comparative Zoology of Harvard University, Museum d’Histoire Naturelle, Museo Civico di Storia Naturale di Milano, The Museum of Michigan State University, Museum of Vertebrate Zoology of University of California, Museo de Zoología de la Universidad Tecnológica Indoamérica, Museo de Zoología de la Pontificia Universidad Católica del Ecuador, Royal Ontario Museum, Santa Barbara Museum of Natural History, Texas Cooperative Wildlife Collection, Museum of Zoology of University of Michigan, Natural History Museum of Utah, National Museum of Natural History, William E. Duellman field series, Western Foundation of Vertebrate Zoology, Yale Peabody Museum of Natural History, Zoologisch Museum Universiteit van Amsterdam, and Zoologisk Museum Universitets København.

1. **Species distribution models**

Based on studies about Maxent performance with low sample sizes (Pearson *et al.*, 2007; Wisz *et al.*, 2008), only species with five or more data records were modeled. The number of background points was set to 10,000 and drawn randomly across all the extension of continental Ecuador. . While using the same background for species from different geographic origins may artificially increase AUC (Area Under de Curve) values (Royle *et al.*, 2012), we were not interested in the fine details of the individual models’ accuracy, but rather in creating the best possible models to be used by the site-selection algorithm. Accordingly, we discarded species’ SDMs that had AUC values below 0.7 (Elith & Leathwick, 2007). To reclassify the 0.0–1.0 suitability map into presence/absence areas, we used the Maximum Training Sensitivity Plus Specificity threshold, which minimizes the false-positive errors that may identify reserve areas that do not actually contain the target species (Jiménez-Valverde & Lobo, 2007).

Maxent models were broad and imprecise for 16 species with restricted distributions and few occurrences (<10). Therefore, for these species we used the Gower Distance Metric (GDM) modeling method (Carpenter *et al.*, 1993) implemented in the program Diva-Gis (Hijmans *et al.*, 2004). The GDM method tends to overfit the generated model to the occurrence data (Mateo, 2008), yielding more conservative results compared to those observed with Maxent. The GDM method resulted in predictive maps with values ​​from 0 to 100, which indicate the degree of similarity between one pixel and the most similar point of occurrence in the sample of occurrence points (Mateo, 2008). Presence/absence maps were generated using a threshold value of 95 (Carpenter *et al.*, 1993).

SDM algorithms generally indicate the geographic region with the appropriate set of abiotic factors for each species, but there are other factors influencing their distributions, such as biotic interactions, dispersal abilities, and biographic history (Soberón & Peterson, 2005). Therefore, to obtain more accurate approximations of species distributions, we evaluated the Maxent and Domain models by comparing them with species distributions in the literature (Ridgely & Greenfield, 2007; Tirira, 2007; IUCN, 2011; AmphibiaWeb, 2012). When necessary, we removed areas of over-prediction and areas beyond geographic barriers that are likely to preclude species dispersal.

1. **Conservation Goals**

Ideally, species conservation goals should be established for each species independently, using biological and ecological arguments to identify the minimum viable population size [38]. Since biological information of Neotropical species is usually limited or absent, we established species conservation goals between 15% and 30% of each species distribution. To assign a specific proportion of species distributions that should be protected, we applied a priority exercise based on four criteria. Each criterion was scored 1–3, where 1 was the lowest and 3 the highest priority value.

Extinction risk: To assess the value of this criterion for each species, we consulted The International Union for Conservation of Nature Red List of Threatened Species (IUCN, 2011) and the following national red lists: Ron *et al.* (2008) for amphibians, Granizo *et al.* (2002) for birds, Tirira (2011) for mammals, and Valencia *et al.* (2000) for plants. Species classified as vulnerable, endangered, and critically endangered were assigned a score of 3. Species classified as near threatened (NT) were assigned a score of 2, which was the value assigned to species classified as not evaluated (NE) and data deficient (DD), since they could be under some level of threat. Finally, species classified as least concern (LC) received a score of 1. Maximum values assigned to each species correspond to the highest category of threat indicated by the national or global red list category.

Taxonomic uniqueness: Usually, a phylogenetically unique taxon has a higher value for biodiversity conservation than a taxon that is closely related to many other taxa (Vane-Wright *et al.*, 1991). The most reliable way to quantify this evolutionary significance accurately is using a phylogeny for estimating the genetic divergence of all taxa from its relatives and the number of lineages at each node (Isaac *et al.*, 2007). However, for most species and their relatives, phylogenetic relationships are not well resolved. Therefore, we based the uniqueness assessment on the number of species per genus, following previous prioritization exercises (Daniels *et al.*, 1991; Arponen, 2012). The number of species per genus was scaled between 1 and 3. Species belonging to relatively large genera were assigned a score of 1, whereas those belonging to small genera were assigned a score of 3. This categorization was made for each group separately (amphibians, birds, mammals, and plants) to avoid potential taxonomic biases among groups. For this assessment, we used the following sources: Frost (2011) for amphibians, BirdLife International (2011) for birds, IUCN (2011) for mammals, and The Plant List (2011) for plants.

Geographic extension: Although species with restricted distributions may be locally abundant, their limitation to certain habitats or areas could make them more susceptible to potential extinctions (Purvis *et al.*, 2000). The extents of species distributions were scaled between 1 and 3; species with largest extensions were assigned a score of 1, whereas species with the smallest distributions were assigned a score of 3. Similar to the assigning of taxonomic uniqueness, this process was conducted for each group separately, especially because plants evaluated in this study usually had large distributions compared to animal species.

Dispersion and home range*:* Small home range and limited dispersion are biological attributes that predispose species to extinction because localized disturbances can result in local extinctions, and if a local extinction occurs, the likelihood of recolonization is low (Blaustein *et al.*, 1994). Amphibians tend to have small home ranges and extremely limited daily movements, compared to other small tretapods and birds, and therefore their recolonization rates are low (Blaustein *et al.*, 1994; Elmer *et al.*, 2007). Species with small home ranges and low dispersion, in this case, all amphibians, were assigned a score of 3, whereas species with largest home range (e.g., plants) or high dispersion capabilities (e.g., birds and mammals) were assigned a score of 1.

For each species, a combined priority score was calculated by summing the scores assigned for the four criteria. Therefore, the maximum priority value that a species could reach was 12, and the lowest value was 4. Species conservation goals were obtained in a linear scale from 15%, assigned to species with the lowest conservation priority, up to 30%, assigned to species with the highest priority.

1. **Environmental Risk Surface**

The ERS is created by combining information about the intensity and distance of influence of environmental risks. A risk element may be defined as any aspect that has a negative influence on the conservation feature. The intensity value summarizes the relative level of threat that the risk element poses to the biodiversity on a 0–100 scale, with 100 being the strongest risk. The influence distance represents the maximum distance at which the element has a negative impact on biodiversity. Within this distance we chose a linear decreasing function to simulate the decline of intensity starting at the location of the risk element.

Based on the available data, we selected the following risk elements: human population density, agriculture and cattle farming, mining and oil concessions, oil wells, dams, roads, and airports (Table 1). Each risk element was assigned an intensity value and an influence distance based in the literature and our judgment. This process yielded a raster layer where each pixel had an impact value between 0–100. The impact values for all pixels in a planning unit were summed to inform Marxan analyses. Additionally, we reduced 40% of the environmental impact in all planning units included within Protected Forests and 10 km around all protected areas. These reductions are justified by the lower cost of acquisition and management that these areas would have in the scenario of creating new protected areas, because they already have a certain level of protection. Also, historically in Ecuador, the process of creating new protected areas usually involves upgrading the level of protection for an already existing conservation area; for example the Yacurí National Park, created in 2009, was previously a Protected Forest (Sánchez *et al.*, 2009)

This factor generates an additional cost in case of failure to reach the conservation goal for a given species (Ardron *et al.*, 2008). Therefore, high PF values ​​guides Marxan into selecting PUs that allow achieving the defined species conservation goals.

**5. Conservation priority and feasibility**

We evaluated priority conservation using two variables. The first was the importance of the area to achieve an efficient reserve system, calculated as the sum of the selection frequency of each PU in the area, divided by its size. The second variable was the environmental impact of the area, and therefore the vulnerability to lose its species diversity, calculated as the summation of ERS across PUs included in the area, divided by its total extension. Then, we used a contingency table to classify the new areas in four groups according to the relationship between two levels of two variables: high or low importance, and high or low environmental impact. We used the Jenks optimization method to arrange these values, because this method seeks to reduce the variance within the two classes and maximize the variance between them [74]. We combined the two classes obtained by each area, to determine its conservation priority:

Maximum priority: Areas with high importance and high environmental impact. Urgent efforts are necessary to avoid losing areas that are vulnerable but important to achieve species conservation goals.

High priority: Areas with low importance but high environmental impact. Measures are necessary to avoid losing areas that are not highly efficient, but contribute to achieve the species conservation goals.

Medium priority: Areas with high importance but low environmental impact. Protection of these areas is less urgent, but constant monitoring is recommended.

Low priority: Areas with low importance and low environmental impact.

The feasibility assessment was also conducted using two variables. The first variable was the proportion of a potential conservation area within Protected Forests and buffer zones of 10 km around reserves. Areas with high values of this variable are more likely to be preserved because efforts for their conservation are on place, and therefore may have lower costs of acquisition, establishment, and management (i.e. public Protectected Forest). The second variable was the proportion of remaining natural vegetation in the new area. Areas with low proportion of remaining natural vegetation require ecological restoration to make them suitable for biodiversity conservation. This restoration will demand more economic and time investments, making these areas less feasible for conservation. As with the priority assessment, the values obtained by each area were classified in four groups using a contingency table (high or low proportion with Protected Forest and buffer zones, and high or low proportion of natural vegetation) defined using Jenks optimization method [74]:

Maximum feasibility: Areas with a high proportion of land within Protected Forests and buffer zones, and conserving a high proportion of natural vegetation.

High feasibility: Areas with a low proportion of land within Protected Forest and buffer zones, but still conserving a high proportion of natural vegetation. They have high feasibility because they still hold suitable environment for conservation, reducing the investment in management.

Medium feasibility: Areas with a high proportion of land within Protected Forests and buffer zones, and a low proportion of natural vegetation conserved. They have medium feasibility because, despite their location in Protected Forests and buffer zones, their implementation may require ecological restoration.

Low feasibility: Areas with a low proportion of land within Protected Forests and buffer zones, and little natural vegetation remaining. They have low feasibility because there are no previous efforts to facilitate their conservation, and thus high investments are needed to restore natural vegetation.

**References**

AmphibiaWeb (2012) *Information on amphibian biology and conservation*. Available at: http://amphibiaweb.org/ (accessed 15-Sep-2012).

Ardron, J.A., Possingham, H.P. & Klein, C.J. (2008) *Marxan Good Practices Handbook*. Pacific Marine Analysis and Research Association, Vancouver.

Arponen, A. (2012) Prioritizing species for conservation planning. *Biodiversity and Conservation*, 21, 875-893.

BirdLife International (2011) *IUCN Red List for birds*. Available at: www.birdlife.org (accessed 15-Sep-2012).

Blaustein, A.R., Wake, D.B. & Sousa, W.P. (1994) Amphibian declines: judging stability, persistence, and susceptibility of populations to local and global extinctions. *Conservation Biology*, 8, 60-71.

Carpenter, G., Gillison, A.N. & Winter, J. (1993) DOMAIN: a flexible modeling procedure for mapping potential distributions of plants and animals. *Biodiversity and Conservation*, 2, 667-680.

Daniels, R., Hedge, M., Joshi, N.V. & Gagdil, M. (1991) Assigning Conservation Value: A Case Study from India. *Conservation Biology*, 5, 464-475.

Elith, J. & Leathwick, J. (2007) Predicting species distributions from museum and herbarium records using multiresponse models fitted with multivariate adaptive regression splines. *Diversity and Distributions*, 13, 265-275.

Elmer, K.R., Davila, J.A. & Lougheed, S.C. (2007) Applying new inter-individual approaches to assess fine-scale population genetic diversity in a neotropical frog, *Eleutherodactylus ockendeni*. *Heredity (Edinb)*, 99, 506-515.

Frost, D.R. (2011) *Amphibian Species of the World: an Online Reference. Version 5.5*. Available at: http://research.amnh.org/vz/herpetology/amphibia/ (accessed 15-Sep-2012).

Granizo, T., Pacheco, C., Ribadeneira, M.B., Guerrero, M. & Suárez, L. (2002) *Libro rojo de las aves del Ecuador*. SIMBIOE, Conservación Internacional, EcoCiencia, Ministerio del Ambiente y UICN, Quito.

Hijmans, R.J., Guarino, L., Bussink, C., Mathur, P., Cruz, M., Barrantes, I. & Rojas, E. (2004) *DIVA-GIS, version 4. A geographic information system for the analysis of biodiversity data. Manual*. Available at: www.diva-gis.org.

Isaac, N.J.B., Turvey, S.T., Collen, B., Waterman, C. & Baillie, J.E.M. (2007) Mammals on the EDGE: Conservation priorities based on threat and phylogeny. *PLoS ONE*, 2, e296.

IUCN (2011) *IUCN Red List of Threatened Species. Version 2011.1*. Available at: www.iucnredlist.org. (accessed 9-Nov-2011).

Jiménez-Valverde, A. & Lobo, J.M. (2007) Threshold criteria for conversion of probability of species presence to either–or presence–absence. *Acta Oecologica*, 31, 361-369.

Mateo, R.G. (2008) *Modelos predictivos de riqueza de diversidad vegetal. Comparación y optimización de métodos de modelado ecológico*. PhD Thesis, Universidad Complutense de Madrid, Madrid.

Pearson, R.G., Raxworthy, C.J., Nakamura, M. & Townsend Peterson, A. (2007) Predicting species distributions from small numbers of occurrence records: a test case using cryptic geckos in Madagascar. *Journal of Biogeography*, 34, 102-117.

Purvis, A., Gittleman, J.L., Cowlishaw, G. & Mace, G.M. (2000) Predicting extinction risk in declining species. *Proceedings of the Royal Society of London B*, 267, 1947-1952.

Ridgely, R. & Greenfield, P.J. (2007) *Aves del Ecuador. Volumen II Guía de Campo*. Fundación Jocotoco, Quito.

Ron, S.R., Guayasamin, J.M., Coloma, L.A. & Menéndez-Guerrero, P. (2008) *Lista Roja de los Anfibios de Ecuador*. Available at: www.puce.edu.ec/zoologia/sron/roja/ (accessed 2011).

Royle, J.A., Chandler, R.B., Yackulic, C. & Nichols, J.D. (2012) Likelihood analysis of species occurrence probability from presence-only data for modelling species distributions. *Methods in Ecology and Evolution*, 3, 545-554.

Sánchez, O.A., Toro, J. & Valle, D.R. (2009) *Plan de Manejo del Área de Conservación Colombo Yacurí*. Fundación Ecológica Arcoiris & Ministerio del Ambiente del Ecuador, Loja.

Soberón, J. & Peterson, A.T. (2005) Interpretation of models of fundamental ecological niches and species’ distributional areas. *Biodiversity Informatics*, 2, 1-10.

The Plant List (2011) *Version 1.* Available at: http://www.theplantlist.org (accessed 15-Sep-2012).

Tirira, D. (2007) *Guía de Campo de los mamíferos del Ecuador*. Ediciones Murciélago Blanco, Publicación especial sobre los mamíferos del Ecuador 6, Quito.

Tirira, D. (2011) *Libro Rojo de los mamíferos del Ecuador. 2a edición. Versión 1.* Fundación Mamíferos y Conservación, Pontificia Universidad Católica del Ecuador y Ministerio del Ambiente del Ecuador, Quito.

Valencia, R., Pitman, N., León-Yánez, S. & Jorgensen, P.M. (2000) *Libro Rojo de las Plantas Endémicas del Ecuador 2000*. Publications of QCA Herbarium, Pontificia Universidad Católica del Ecuador, Quito.

Vane-Wright, R.I., Humphries, C.J. & Williams, P.H. (1991) What to Project? – Systematics and the agony of choice. *Biological Conservation*, 55, 235–254.

Wisz, M.S., Hijmans, R.J., Li, J., Peterson, A.T., Graham, C.H. & Guisan, A. (2008) Effects of sample size on the performance of species distribution models. *Diversity and Distributions*, 14, 763-773.

**Table 1. Intensity and influence distances assigned to different risk elements used to create the environmental risk surface.**

| **Risk element** | **Class** | **Intensity**  **(0–100)** | **Influence distance** |
| --- | --- | --- | --- |
| Roads | 4-lane highway | 50 | 1,000 m |
|  | 2-lane highway | 25 |  |
|  | Track | 17 |  |
|  | Railroad | 17 |  |
|  | Dirty roads | 10 |  |
| Human population density | – | Normalized between 0 and 100 according to the logistic function | Scaled from 5 km from the lower intensity |
| Airports | – | 70 | 2,000 m |
| Dams | – | 50 | 1,000 m |
| Agriculture and cattle raising | Monoculture plantations and cattle raising | 50 | 2,000 m |
|  | Mixed crops | 40 | 1,000 m |
|  | Forestry crops and crops in protected forests | 30 | 1,000 m |
|  | Shrimp farms | 50 | 2,000 m |
| Oil industry | Oil wells | 70 | 3,000 m |
|  | Oil concessions | 30 | 3,000 m |
| Mining industry | Mining concessions in exploitation | 50 | 10,000 m |
